# Withdrawal of Continuous Positive Airway Pressure Therapy after Malar Advancement and Le Fort II Distraction in a Case of Apert Syndrome with Obstructive Sleep Apnea

**DOI:** 10.1155/2015/125023

**Published:** 2015-09-14

**Authors:** Nobuto Onda, Shintaro Chiba, Hiroto Moriwaki, Rika Sawai, Akira Yoshigoe, Subaru Watanabe, Yuji Ando, Ryo Uchida, Takeshi Miyawaki, Kota Wada

**Affiliations:** ^1^Department of Otorhinolaryngology, The Jikei University School of Medicine, 3-25-8 Nishi-Shimbashi, Minato-ku, Tokyo 105-8461, Japan; ^2^Department of Plastic and Reconstructive Surgery, The Jikei University School of Medicine, Tokyo, Japan; ^3^Department of Otorhinolaryngology, Toho University, Tokyo, Japan

## Abstract

Apert syndrome is a congenital syndrome characterized by craniosynostosis and craniofacial dysostosis, among other features, and is reported to cause obstructive sleep apnea (OSA) because of upper airway narrowing associated with midfacial dysplasia. We recently encountered a case involving a patient with Apert syndrome complicated by OSA who began to receive continuous positive airway pressure (CPAP) therapy at the age of 4. OSA resolved after maxillofacial surgery performed at the age of 11, and CPAP was eventually withdrawn. In pediatric patients with maxillofacial dysplasia complicated by OSA, a long-term treatment plan including CPAP in addition to maxillofacial plastic and reconstructive surgery should be considered in view of the effects of OSA on growth.

## 1. Introduction

Apert syndrome is a congenital syndrome characterized by craniosynostosis, craniofacial dysostosis, and symmetrical syndactyly in the hands and feet. Fibroblast growth factor receptor 2 (FGFR2) is reportedly the responsible gene [1]. Epidemiologically, the rate of complications associated with sleep dyspnea, including OSA, ranges from 40% to 80%, and no consensus has been reached regarding the same.

This syndrome has been reported to cause obstructive sleep apnea (OSA) because of upper airway narrowing associated with midfacial dysplasia [2]. Although several studies have reported on craniofacial abnormalities, few have objectively discussed the management of sleep-related breathing disorders in these patients [3].

The general therapeutic strategy for pediatric OSA differs depending on the presence or absence of pharyngeal tonsil or palatine tonsil hypertrophy. When hypertrophy is present, the first-line therapy is adenoidectomy or tonsillectomy, except in patients who are contraindicated to surgical resection such as morbid obesity, refractive hemorrhagic diseases, and submucosal cleft palate. When hypertrophy is not present or when patients are contraindicated to surgery, other modalities such as nasal steroid spray and CPAP should be considered. With regard to the use of nasal steroid spray, the treatment efficacy varies among individuals. Furthermore, no studies have evaluated the time to efficacy; therefore, efficacy should be evaluated after a course of approximately 6 weeks, while the presence or absence of relapse or adverse events related to steroid should be observed through follow-up visits [4]. With regard to CPAP therapy, the appropriate pressure differs among individuals and over time; therefore, it should be determined by titration before prescription, with periodic readjustments. In addition, the fit of the head gear and mask should be adjusted as the child grows. Furthermore, the guardians' cooperation is essential because pediatric patients may lack an understanding of subjective symptoms and the necessity to use the equipment. If pediatric patients experience discomfort during attachment to the CPAP equipment in the early stages, they and their family will continue to experience a feeling of rejection for a long time [2]; therefore, special environmental considerations and ways for easy acceptance of CPAP by children are necessary [5, 6].

The general treatment course for Apert syndrome is as follows. First, frontoorbital advancement is performed to prevent an increase in intracranial pressure, mostly at or less than 1 year of age. Then, as the bones grow, Le Fort osteotomy (type depends on individual cases) is performed for an improvement in appearance at approximately 5 years of age. Subsequently, Le Fort III osteotomy or other procedures are performed to achieve stable occlusion after the completion of orthodontic treatment, when the patient has crossed the adolescent stage.

Here we report a case involving a patient with Apert syndrome and OSA in whom CPAP, which was initiated at the age of 4, was withdrawn after the resolution of OSA following maxillofacial surgery at the age of 11.

## 2. Case Presentation

**Figure 1:**
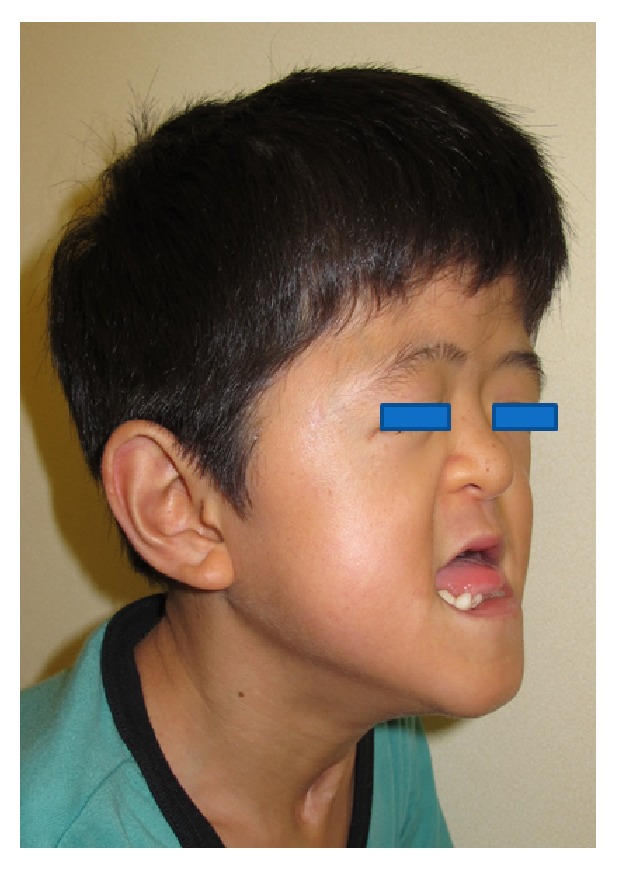
Appearance before surgery.

**Figure 2:**
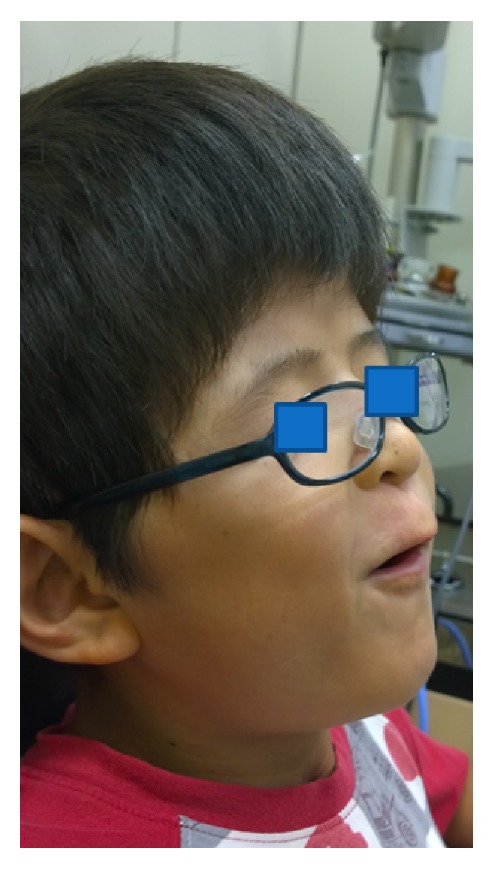
Appearance after surgery. Maxillary hypoplasia, because of which the mouth remained constantly open, and malocclusion have been corrected after surgery.

The patient was a boy born by normal vaginal delivery at the gestational age of 39 weeks. Although both his parents and brother did not have any specific abnormalities, he showed craniosynostosis and syndactyly at birth and was diagnosed with Apert syndrome. He required repeated hospitalization because of several episodes of impaired suckling, vomiting, aspiration pneumonia, and tracheal intubation. Therefore, the patient was referred to the Department of Pediatric Surgery at The Jikei University Hospital for treatment of the complicated malformations and for tracheotomy. Subsequently, cranioplasty and tracheotomy were performed at 1 year and 8 months of age. At the age of 2 and 1 month, left diaphragm plication, cardioplasty, gastropexy, and gastrostomy were performed for the treatment of gastric volvulus, which was diagnosed on detailed investigation of repeated vomiting. Cleft palate repair was also performed. No major airway trouble occurred after surgery; however, the patient removed the tracheal cannula by himself at 3 years and 7 months of age. Fortunately, his respiratory condition remained stable after cannula removal. Therefore, the tracheotomy fistula was surgically closed. Subsequently, however, impaired and laborious respiration and decreased oxygen saturation (SpO_2_, 85%–88%) were observed at night. Overnight polysomnography (PSG) performed at The Ota Sleep Center at 3 years and 10 months of age revealed an apnea-hypopnea index (AHI) of 38.4/h, leading to a diagnosis of severe OSA. Therefore, at 4 years of age, the boy was hospitalized for 3 days for manual titration. The parents were also educated about CPAP therapy during this time. Because the priority of the test was titration, it was performed without any equipment other than the SpO_2_ monitor. The mask was attached after sleep onset, and the test was initiated at 4 cm H_2_O. When the pressure was increased to 5 cm H_2_O, the patient woke up and became violent because of pain. Subsequently, nasal sprays were administered to facilitate nasal breathing; however, mouth breathing persisted and nasal breathing was difficult. Therefore, the patient's condition was observed for a while without using the CPAP. His SpO_2_ decreased to approximately 90% at sleep onset; consequently, the mask was attached again. Then, the pressure could be increased to 8 cm H_2_O without any problems, and nasal breathing became possible when his mother held his chin, while his SpO_2_ remained stable at ≥97%. On the basis of these results, CPAP therapy with a fixed pressure of 9 cm H_2_O was prescribed. After the introduction of CPAP, his respiration became less laborious. CPAP compliance was also good. According to his mother, tube feeding through the gastrostomy soon became unnecessary because of increased food intake; furthermore, his growth rate showed an improvement. At 10 years of age, maxillofacial surgery (malar advancement and Le Fort II distraction) was performed at The Department of Plastic and Reconstructive Surgery in The Jikei University Hospital to enlarge the upper airway, improve his facial appearance, correct malocclusion, and resolve exophthalmos, as treatments for Apert syndrome (Figures 1 and 2) [7, 8]. The reason that we chose Le Fort II osteotomy not Le Fort III osteotomy is because the distance to correct malocclusion, the distance to improve the figure of external nose, and the distance to resolve exophthalmos are different, respectively [9].

After the surgery, CPAP was withdrawn because of the effect of external fixation, and the patient's condition was observed through follow-up visits. His symptoms of OSA were barely observed. On day 35 after surgery, the extension device was removed and PSG was performed to reevaluate OSA. The results at the age of 11 and 5 months were markedly improved, with AHI value of 7.5/h. In addition, oxygen saturation and the sleep stage structure showed a marked improvement (Table 1). On the basis of these results and at the request of the patient's family, CPAP was withdrawn.

## 3. Discussion

The present patient exhibited Apert syndrome complicated by OSA. According to his mother, after the initiation of CPAP therapy, tube feeding through the gastrostomy became unnecessary because his oral intake improved; furthermore, his growth rate increased. These improvements may be attributed to the resolution of OSA by CPAP therapy. Consequently, his sleep-dependent metabolism, including the secretion of growth hormones, may have improved. However, there are no objective data to support these speculations.

**Figure 3:**
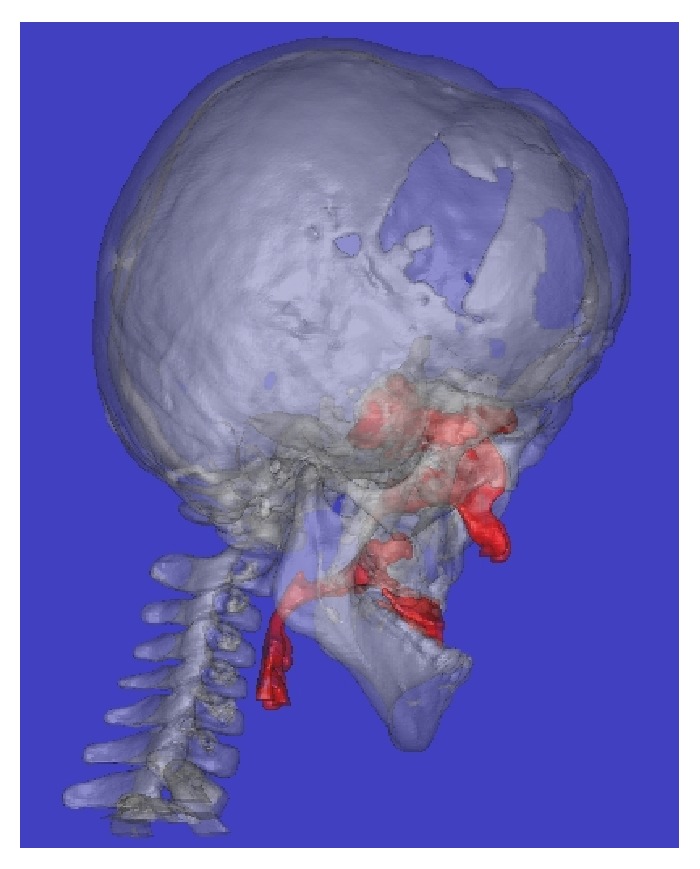
A side view of a three-dimensional computed tomography image obtained before surgery.

**Figure 4 fig4:**
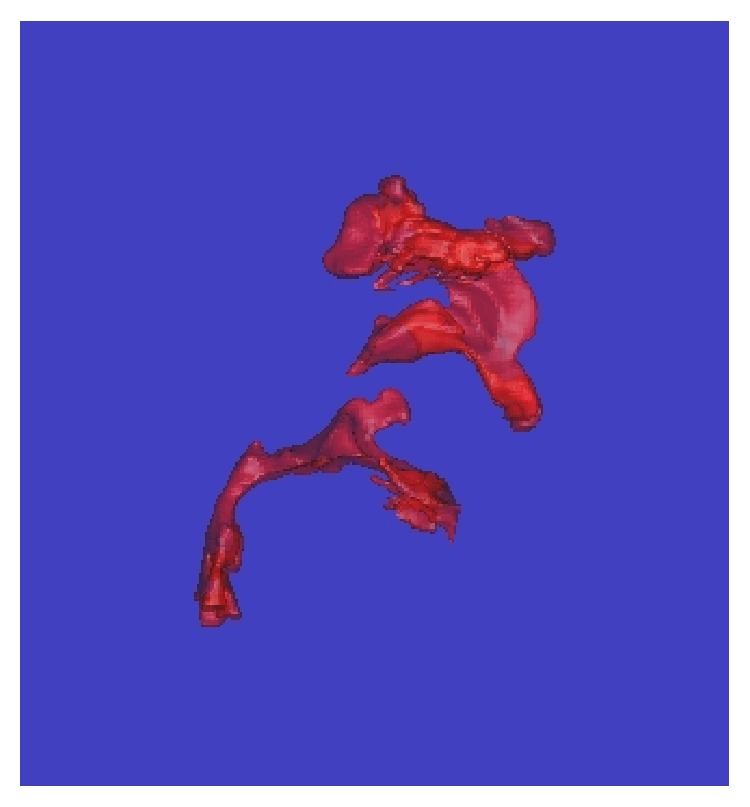
Three-dimensional computed tomography of the airway before surgery.

**Figure 5 fig5:**
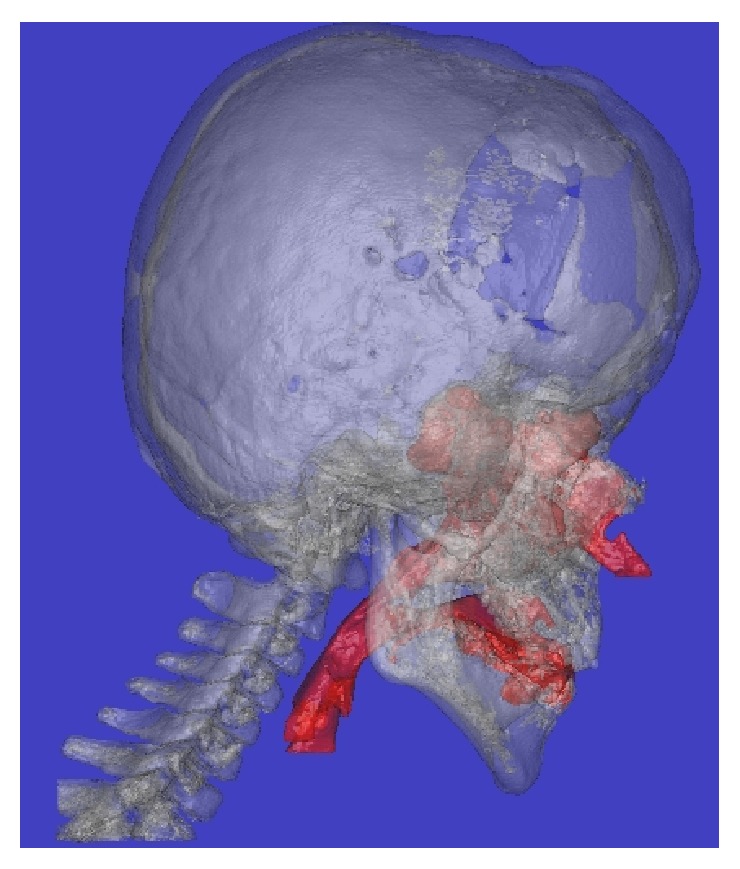
A side view of a three-dimensional computed tomography image obtained after surgery. Maxillary hypoplasia is mostly rectified compared with that before surgery.

**Figure 6:**
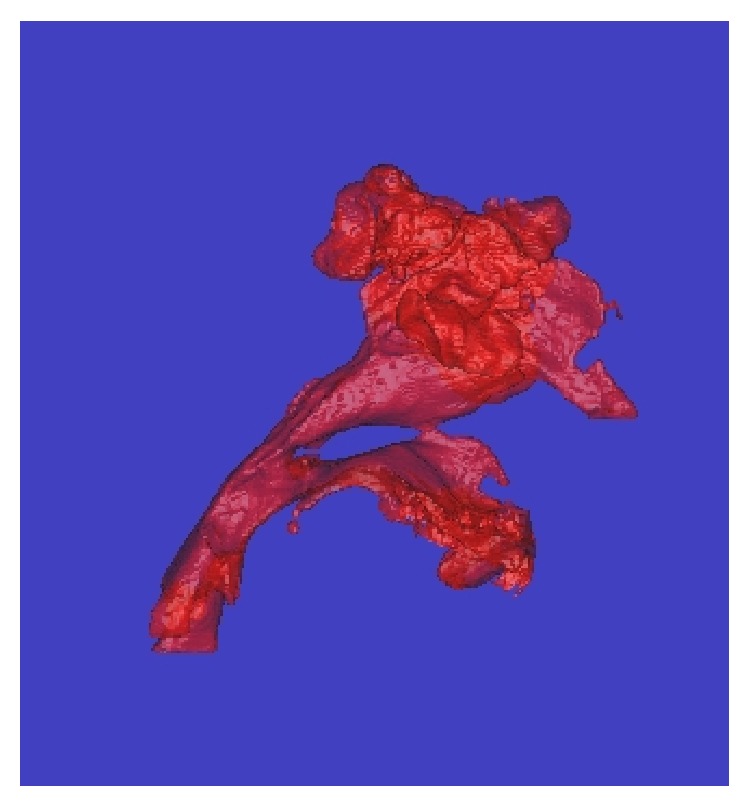
Three-dimensional computed tomography of the airway after surgery. The volume of the airway around the pharyngeal cavity is markedly increased compared with that before surgery.

The actual extension distance associated with surgery is expressed as the measured moving distance of point A on a lateral cephalogram from immediately before surgery to immediately after removal of the extension device (Table 2). Because the sella and nasion did not change before and after surgery, these two points were used as reference points. Then, a line connecting the sella and nasion (S-N line) and a line passing through the nasion that was perpendicular to the S-N line were drawn (the plane containing these two lines is hereafter referred to as the facial plane). When the moving distance of point A was measured using these two lines as a frame of reference, the moving distance along the S-N line was 8 mm toward the nasion, while that along the facial plane was 10.9 mm caudally. Therefore, the linear moving distance of point A was 13.5 mm. In addition, when the airway volumes were measured using three-dimensional computed tomography (3D-CT) immediately before and 6 months after surgery, the pharyngeal cavity rose about sixfold from 9187 mm^3^ to 59270 mm^3^ (Figures 3 [Fig fig4] [Fig fig5]–6). Therefore, the factors that enabled the withdrawal of CPAP at 11 years of age, after its introduction at 4 years of age, may be considered to be airway enlargement following maxillofacial plastic and reconstructive surgery and the subsequent resolution of OSA [10, 11].

## 4. Conclusions

In conclusion, we reported a case of Apert syndrome complicated by OSA, in which growth and development were supported by CPAP for almost 7 years before plastic and reconstructive surgery. After the surgery, OSA resolved and CPAP could be withdrawn. In pediatric patients with maxillofacial dysplasia complicated by OSA, a long-term treatment plan including CPAP should be considered in view of the effects of OSA on growth.

## Figures and Tables

**Table 1:** Overnight polysomnography (PSG) before and after surgery.

	Pre-op	Post-op
TST	474 min	470 min
Sleep latency	4.5 min	4.5 min
Rem latency	68 min	203 min
Sleep efficacy	80.3%	97.8%
%stage 1	29.3%	4.8%
%stage 2	22.9%	33.4%
%stages 3 + 4	33%	46.0%
%stage REM	14.8%	15.8%
AHI/AI	38.4/8.7	7.5/1.0
OA	69	7
CA	0	1
OH	234	51
OD < 90%	18.3%	0%
Lowest SpO_2_	67%	93%

**Table 2:** Lateral cephalogram before and after surgery.

	Pre-op	Post-op
Facial axis	75.3°	79.0°
SNA	61.0°	84.5°
SNB	73.8°	79.4°
MPH	15.2 mm	12.7 mm
PAS	7.8 mm	12.1 mm

## References

[1] Fearon J. A., Podner C. (2013). Apert syndrome: evaluation of a treatment algorithm. *Plastic and Reconstructive Surgery*.

[2] Driessen C., Joosten K. F. M., Bannink N. (2013). How does obstructive sleep apnoea evolve in syndromic craniosynostosis? A prospective cohort study. *Archives of Disease in Childhood*.

[3] Arens R., Marcus C. L. (2004). Pathophysiology of upper airway obstruction: a developmental perspective. *Sleep*.

[4] Marcus C. L., Brooks L. J., Draper K. A. (2012). Diagnosis and management of childhood obstructive sleep apnea syndrome. *Pediatrics*.

[5] Koontz K. L., Slifer K. J., Cataldo M. D., Marcus C. L. (2003). Improving pediatric compliance with positive airway pressure therapy: the impact of behavioral intervention. *Sleep*.

[6] Rains J. C. (1995). Treatment of obstructive sleep apnea in pediatric patients: behavioral intervention for compliance with nasal continuous positive airway pressure. *Clinical Pediatrics*.

[7] McCarthy J. G., Schreiber J., Karp N., Thorne C. H., Grayson B. H. (1992). Lengthening the human mandible by gradual distraction. *Plastic and Reconstructive Surgery*.

[8] Matsumoto K., Nakanishi H., Koizumi Y. (2002). Segmental distraction of the midface in a patient with Crouzon syndrome. *Journal of Craniofacial Surgery*.

[9] Hopper R. A., Kapadia H., Morton T. (2013). Normalizing facial ratios in apert syndrome patients with le fort ii midface distraction and simultaneous zygomatic repositioning. *Plastic and Reconstructive Surgery*.

[10] Wada K., Moriwaki H., Ota F., Chiba S. (2005). A case study of combined apert and sleep-apnea syndrome. *JIBI INKOKA TEMBO*.

[11] Mitsukawa N., Kaneko T., Saiga A., Akita S., Satoh K. (2013). Early midfacial distraction for syndromic craniosynostotic patients with obstructive sleep apnoea. *Journal of Plastic, Reconstructive & Aesthetic Surgery*.

